# Physiochemical Studies of Sodium Chloride on Mungbean (*Vigna radiata* L. Wilczek) and Its Possible Recovery with Spermine and Gibberellic Acid

**DOI:** 10.1155/2015/858016

**Published:** 2015-02-03

**Authors:** Srijita Ghosh, Sanglap Mitra, Atreyee Paul

**Affiliations:** Department of Botany, Scottish Church College, 1 and 3 Urquhart Square, Kolkata 700006, India

## Abstract

The physiological and biochemical responses to increasing NaCl concentrations, along with low concentrations of gibberellic acid or spermine, either alone or in their combination, were studied in mungbean seedlings. In the test seedlings, the root-shoot elongation, biomass production, and the chlorophyll content were significantly decreased with increasing NaCl concentrations. Salt toxicity severely affected activities of different antioxidant enzymes and oxidative stress markers. Activities of antioxidant enzymes, superoxide dismutase (SOD), and catalase (CAT) increased significantly over water control. Similarly, oxidative stress markers such as proline, malondialdehyde (MDA), and hydrogen peroxide (H_2_O_2_) contents also increased as a result of progressive increase in salt stress. Combined application of NaCl along with low concentrations of either gibberellic acid (5 *µ*M) or spermine (50 *µ*M) in the test seedlings showed significant alterations, that is, drastic increase in seedling elongation, increased biomass production, increased chlorophyll content, and significant lowering in all the antioxidant enzyme activities as well as oxidative stress marker contents in comparison to salt treated test seedlings, leading to better growth and metabolism. Our study shows that low concentrations of either gibberellic acid or spermine will be able to overcome the toxic effects of NaCl stress in mungbean seedlings.

## 1. Introduction

Increase in the world population and the decreasing trend of arable land has led to a marked decrease in food security with abiotic stresses, salinity being one of the major contributors in decreasing the crop productivity. Nearly 800 million hectares of land all over the world (6% of the world's total land area) are salt affected [1]. Salinization problems are on the increase mainly due to poor irrigation drainage or agricultural practices [2]. This drastic increase in land area being affected by salinity urges the need to develop strategies to enhance crop productivity under saline conditions. The negative effects of salinity, owing to increase in Na^+^ and Cl^−^ ions (with Cl^−^ being more dangerous), disturb the homeostasis of essential nutrients [3–5], leading to both hyperionic and hyperosmotic stress. The effects may be membrane damage, nutrient imbalance, alterations in levels of growth regulators, enzymatic inhibition, reactive oxygen species (ROS) generation leading to DNA damage, and activation of programmed cell death [6–9]. Plants tend to develop multiple detoxification mechanisms to prevent ROS from damaging cellular components [10].

Phytohormones interact with nutrients synergistically or antagonistically and regulate plant growth and development under optimal and stressful environments. Different phytohormones such as cytokinins (CK) [11], auxins [12], gibberellins (GA) [13], ethylene [14], nitric oxide (NO) [15], jasmonates (JA) [16], and salicylic acid (SA) [17] play essential roles in alleviating salt stress by regulating plant growth and development. Gibberellic acid (GA_3_) ameliorates harmful impacts of salinity [13, 18] by establishing better seedling growth and shoot-root biomass. GA_3_ favorably affects the water status of the seedlings by partially sustaining protein and RNA levels. Exogenous GA_3_ application increased length and dry mass and reduced oxidative stress marker proline in salt affected soybean plants. It also maintained normal growth and development and reversed the inhibitory effects of salt on germination and seedling development in* Arabidopsis*. GA_3_ induces salt tolerance in plants by increasing sucrose, reducing sugar contents, the protein synthesis machinery, and the activity of antioxidant enzymes [18–20].

Polyamines (PAs) are small, low molecular weight, nonproteinaceous, straight chain, aliphatic hydrocarbon compounds with amino and imino groups. They are positively charged, organic molecules that are ubiquitous in all living organisms. The three common PAs in plants are putrescine (Put), spermidine (Spd), and spermine (Spm), with some plants also having thermospermine (tSpm) in place of or in addition to Spm. They have been deemed important in preparing the plant for stress tolerance and to directly aid in ameliorating the causes of stress [21]. Exogenous polyamine application is said to increase endogenous PAs and reverse salt stress damage in vegetative tissues of several plants including rice [22–26]. Exogenous PA application also promotes reproductive development under normal growth conditions and offers protection to reproductive structures against abiotic stress [27]. Application of Put reduces the net accumulation of Na^+^ and Cl^−^ ions in different organs of* Atropa belladonna* subjected to salinity stress. Put increased the growth and the leaf tissue viability of salt treated rice cultivars [22, 27].

Pulses are the best dietary source of proteins and they play a very important role to fulfill the requirements of rapidly increasing population. Mungbean (*Vigna radiata *(L.) Wilczek) is an important, self-pollinated, environment friendly, short season pulse crop which is grown primarily for its protein rich edible seeds. Abiotic stresses severely reduce the productivity of almost all pulses, including mungbean [28]. However, stress-induced adverse effects are variable at various growth stages. In mungbean, the adverse effect on grain yield is more at the reproductive stage than that at other stages. Mungbean is known as a salt sensitive crop [2, 29] and increasing salt concentrations reduce seed germination, fresh and dry biomass, shoot and root lengths, photosynthesis, and yield attributes of mungbean [2, 5, 28, 30].

On this note, the present study is primarily focused to study the toxic effects of salt stress in mungbean and its possible amelioration by GA_3_ or Spm with emphasis on morphological parameters such as overall plant growth, fresh and dry weight, root, shoot, and leaf length, and certain biochemical changes including chlorophyll content, stress induced damage in the form of elevated malondialdehyde (MDA), proline, and hydrogen peroxide (H_2_O_2_) content or the enhanced effect of antioxidant enzymes like catalase (CAT) and superoxide dismutase (SOD).

## 2. Materials and Methods

### 2.1. Plant Material, Growth Conditions, and Stress Treatments

Seeds of mungbean (*V. radiata *L. Wilczek) variety B-105, obtained from Pulse and Oilseed Research Institute, Behrampore, were surface sterilized with 0.1% (w/v) HgCl_2_ for 10 min, washed extensively, and allowed to germinate in Petri dishes lined with water-soaked filter paper at 27 ± 2°C for two days. The germinated seedlings were next transferred to separate Petri dishes containing varying concentrations of NaCl (25 mM, 50 mM, and 100 mM) with or without 5 *μ*M concentration of GA_3_ or 50 *μ*M Spm. Water controls (0 mM NaCl) were run parallel to each experiment. All the Petri dishes were maintained at well aerated places under properly illuminated conditions. Following eight days of stress imposition, NaCl-stressed seedlings were monitored for their overall growth. In addition, the seedlings were washed thoroughly and used for the various biochemical analyses.

### 2.2. Measurement of Root Length and Shoot Length, Fresh Weight and Dry Weight of Seedlings

Following stress imposition of two-day-old germinated seedlings for eight days, about 20 seedlings were randomly selected from each set and washed thoroughly with water. The root, shoot, and leaf length were measured in centimeter scale in five independent experiments.

For calculation of fresh and dry weight, about 20 seedlings were selected for measuring the fresh weight of mungbean seedlings from each set separately. Following fresh weight measurement, the tissues were kept in hot air oven at 50°C for two days followed by three days at 80°C and the weight of dried seedlings was noted.

### 2.3. Estimation of Chlorophyll Content

About 0.5 g of leaf samples harvested from untreated or NaCl-treated seedlings were used for the estimation of chlorophyll content [31]. Chlorophyll was extracted with 80% (v/v) chilled alkaline acetone. The absorbance for chlorophyll b at 645 nm and for chlorophyll a at 663 nm and of total chlorophyll was recorded using the formula [{(20.2 × *A*645)+(8.02 × *A*663)}/1000 × *W*] × *V*, where “*W*” is the fresh weight of the material and “*V*” is the extraction volume and is expressed in terms of *μ*g g^−1^ fresh tissue.

### 2.4. Estimation of Proline Content

The proline content in the untreated or NaCl-treated seedlings was estimated [32]. 0.5 g of tissue was homogenized with 5 mL of 0.1 M sulphosalicylic acid and centrifuged at 5000 ×g. The supernatant was adjusted to 5 mL with distilled water. 2 mL of the extract was incubated with 5 mL of glacial acetic acid and 5 mL of acid ninhydrin and the tubes were heated in boiling water bath for 1 h. Following cooling of the tubes, the samples were extracted with 10 mL of toluene and the pink colour intensity was recorded at 520 nm against a standard curve prepared using known concentrations of proline and expressed in terms of *μ*g g^−1^ fresh tissue.

### 2.5. Estimation of MDA

About 0.5 g of test seedlings was used for MDA assay [33]. The samples were homogenized with 50 mM buffer solution, which contained 0.07% NaH_2_PO_4_·2H_2_O and 1.6% Na_2_HPO_4_·12H_2_O, and centrifuged at 20,000 ×g for 25 min at 4°C. About 4 mL of 20% (v/v) trichloroacetic acid (TCA) containing 0.5% (w/v) thiobarbituric acid was added to 1 mL aliquot of supernatant. The mixture was heated at 95°C for 30 min, quickly cooled on ice, and centrifuged for 10 min. The absorbance of the supernatant was read at 532 nm and the value of nonspecific absorption at 600 nm was subtracted from 532 nm reading. The MDA concentration was calculated using extinction coefficient 155 mM^−1 ^cm^−1^ and expressed in terms of *μ*M g^−1^ fresh tissue.

### 2.6. Estimation of H_2_O_2_ Content

The H_2_O_2_ content in the untreated or NaCl-treated was estimated as described earlier [34]. 0.5 g of plant tissue was homogenized with cold 5% (w/v) TCA at 4°C and the homogenate was centrifuged at 17,000 ×g at 4°C for 10 min. The supernatant was immediately used for estimation of H_2_O_2_ by the ferrothiocyanate method. Each reaction mixture contained 2 mL of the extract, 0.5 mL of 50% TCA solution, 0.5 mL of 10 mM ferrous ammonium sulfate, 0.3 mL of 2.5 M potassium thiocyanate, and 1.7 mL of distilled water. The absorbance of the ferrothiocyanate complex formed was read at 480 nm and was compared with the standard curve prepared with known concentration of H_2_O_2_. H_2_O_2_ content was expressed in terms of *μ*M g^−1^ fresh tissue.

### 2.7. Assay of Catalase Activity

The test seedlings were homogenized in 0.1 M phosphate buffer pH 7.0. The homogenate was centrifuged at 10,000 ×g for 20 min at 4°C. The Catalase (CAT, EC 1.11.1.6) assay was performed following the standard protocol [35] with certain modifications. The standard reaction mixture contained 1 mL of enzyme extract, 10 mL of 0.1 M phosphate buffer pH 7.4, and 1 mL of 5% H_2_O_2_ and the sets were incubated for 30 min at room temperature (25°C). The blank set contained 1 mL of phosphate buffer instead of 1 mL of enzyme extract in a sample set. The reaction was stopped by the addition of 5 mL of 10% H_2_SO_4_. The residual H_2_O_2_ was titrated against 0.02 N KMnO_4_. By estimating the amount of KMnO_4_ consumed in terms of H_2_O_2_ (1 mL of 0.02 N KMnO_4_~17 mg H_2_O_2_), total H_2_O_2_ was calculated. The total soluble protein content of the extract was also determined and the CAT activity was expressed in terms of mg H_2_O_2_ decomposed h^−1 ^mg^−1^ total protein.

### 2.8. Assay of Superoxide Dismutase Activity

The test seedlings were homogenized in 50 mM Tris-HCl buffer pH 7.5 containing 0.1 mM EDTA and 10% polyvinyl pyrrolidone. The homogenate was centrifuged at 10,000 ×g for 20 min at 4°C. The superoxide dismutase (SOD, EC 1.15.1.1) assay was performed following the standard protocol [36] with certain modifications. The standard reaction contained 2.5 mL 80 mM Tris-HCl (pH 8.9) containing 0.12 mM EDTA and 10.8 mM TEMED, 0.1 mL of (3.3 × 10^−9^)% BSA, 0.1 mL 6 mM NBT, 0.1 mL of 0.6 mM riboflavin in 5 mM KOH, and 0.1 mL supernatant. The glass tubes containing the reaction mixtures were exposed to fluorescent light (40 W) at 25°C. The reaction was terminated by turning the light off. The increase in absorbance due to formation of formazon was read at 560 nm, and the enzyme activity was expressed as enzyme units (EU) min^−1 ^mg^−1^ total protein.

### 2.9. Protein Estimation

In all enzyme preparation protein estimation was done accordingly [37] using Bovine Serum Albumin (BSA, Sigma) as standard.

### 2.10. Statistical Analysis

The experiments were carried out in a completely randomized design with five replicates; each replicate comprised a single Petri dish, containing an average of 50 seeds. The data and significant differences among mean values were compared by descriptive statistics (±standard error, SE) followed by Student's *t*-test.

## 3. Results and Discussions

### 3.1. Effect of NaCl Treatment on Seedling Growth and Development

**Figure 1:**
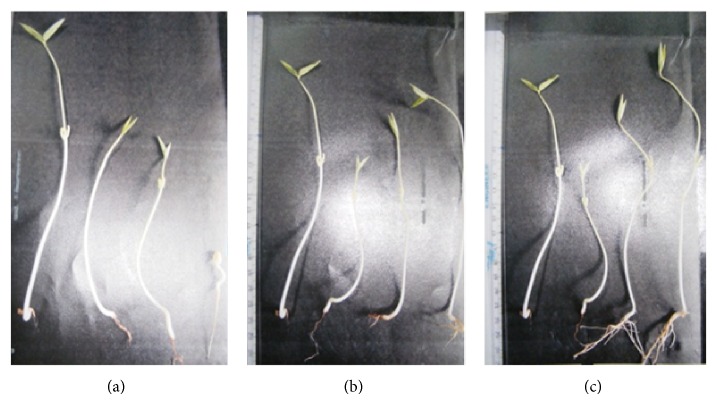
(a) Effect of NaCl on the growth of 10-day-old mungbean seedling. (b) Effect of NaCl on growth of 10-day-old mungbean seedling and its possible amelioration with 5 *μ*M GA_3_. (c) Effect of NaCl on growth of 10-day-old mungbean seedling and its possible amelioration with 50 *μ*M Spm.

**Figure 2:**
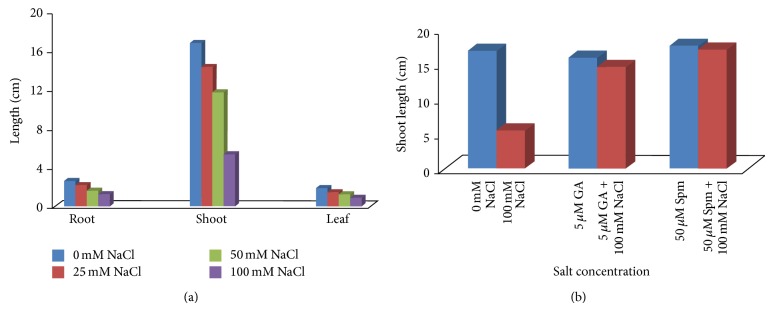
(a) Effect of NaCl on the root, shoot, and leaf length of 10-day-old mungbean seedling. (b) Effect of NaCl on the shoot length of 10-day-old mungbean seedling and its possible amelioration with 5 *µ*M GA_3 _and 50 *µ*M Spm.

Salt treatment showed marked decline in normal growth and development in mungbean seedlings. The extent of retardation enhanced drastically with the progressive increase in salt concentrations [2], the maximum inhibition being at the highest concentration, 100 mM NaCl (Figures 1(a) and 2(a)). Still higher concentrations were not tried as the seedlings failed to survive, showing brownish brittle roots, reddish shoots, and bleached leaves. The inhibitory effect was more pronounced in shoot than in root and leaves. The primary root length was decreased by about 15%, 37%, and 52% in response to 25 mM, 50 mM, and 100 mM NaCl concentrations. In contrast, the shoot lengths on treatment with 25 mM, 50 mM, and 100 mM NaCl concentrations decreased by 15%, 31%, and 68% and leaf lengths by 22%, 34%, and 53%, respectively. When test seedlings were treated jointly with either 5 *μ*M GA_3_ (Figures 1(b) and 2(b)) or 50 *μ*M Spm (Figures 1(c) and 2(b)), the inhibitory effect caused by salt treatment alone was partially relieved. Here 5 *μ*M GA_3_ and 50 *μ*M Spm were standardized by the authors as amelioration dosage. On joint application of 100 mM NaCl with 5 *μ*M GA_3_, the shoot length decreased by 14% over water control, while joint application of 100 mM NaCl with 50 *μ*M Spm showed an increase of 1% over water control. Higher concentrations of either GA_3_ or Spm led to increased oxidative damage in the test seedlings. Also 25 mM and 50 mM NaCl treated test seedlings showed comparatively less damage in respect to water control and hence all further experiments were performed with 100 mM NaCl concentration.

**Figure 3:**
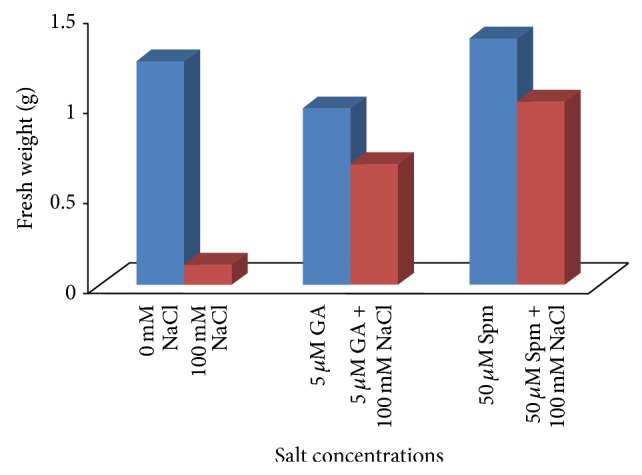
Effect of NaCl on the fresh weight of 10-day-old mungbean seedling and its possible amelioration with 5 *µ*M GA_3_ and 50 *µ*M Spm.

**Figure 4:**
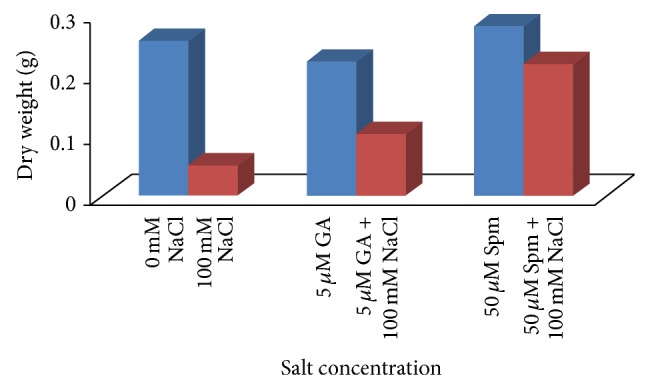
Effect of NaCl on the dry weight of 10-day-old mungbean seedling and its possible amelioration with 5 *µ*M GA_3_ and 50 *µ*M Spm.

The reduction in fresh weight (Figure 3) and dry weight (Figure 4) of seedlings with respect to control was evident with NaCl stress. The fresh weight of seedlings reduced by as much as 92% at 100 mM concentrations over water control. The decrease in dry weight of seedlings was about 80% as compared to water control at the above mentioned NaCl concentrations. Joint application of either 5 *μ*M GA_3_ or 50 *μ*M Spm significantly increased the fresh as well as dry weight of treated samples as compared to direct NaCl treatment. In case of joint application of 100 mM NaCl with 5 *μ*M GA_3_, the fresh and dry weights were decreased by about 45% and 60% over water control, while in case of joint application of 100 mM NaCl with 50 *μ*M Spm, the fresh and dry weights were decreased by about 17% and 15% over water control.

Salinity is known to cause several deleterious morphological effects on different stages of plant growth and development. Growth as well as metabolism is affected under salinity stress [38, 39]. The salinity at seedling stage of cereals causes reduction in germination percentage, fresh and dry weight of shoot and roots [40]. The effects of salinity on plant growth are associated with low osmotic potential and nutrient imbalance. Reduced root and shoot growth in response to salt stress has already been reported in different plant species [2, 5]. Our experiment showed that the root, shoot, and leaf length as well as fresh and dry weights of NaCl-treated seedlings decreased with the increasing NaCl concentration over water control. Decrease in seedling vigour under salinity stress is due to the reduced ability of imbibitions resulting in limited hydrolysis of food reserves from storage tissues. Application of low concentrations of exogenous GA_3_ or Spm was able to reverse the deleterious effects of high salinity stress and allowed normal growth of the seedlings.

### 3.2. Effect of NaCl Treatment on Chlorophyll Content

**Figure 5:**
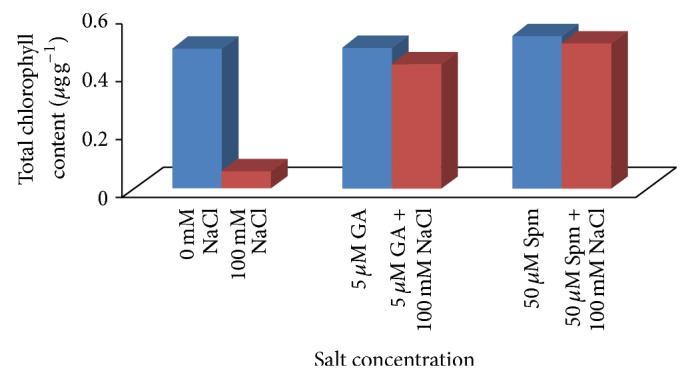
Effect of NaCl on the total chlorophyll content of 10-day-old mungbean seedling and its possible amelioration with 5 *µ*M GA_3 _and 50 *µ*M Spm.

It has already been reported that mungbean plants grown under salinity stress showed excess accumulation of leaf Na^+^ and Cl^−^ resulting in excess reactive oxygen species (ROS) production, reduced photosynthesis, and plant growth [5]. In accordance with previously cited literature, there was a significant decrease in chlorophyll content in 100 mM NaCl treated test seedlings (Figure 5). With respect to water control, the reduction in chlorophyll content was about 89% at 100 mM NaCl concentrations. Joint application of 5 *μ*M GA_3_ or 50 *μ*M Spm significantly increased the total chlorophyll content with respect to control. In case of joint application of 100 mM NaCl with 5 *μ*M GA_3_, the reduction in chlorophyll content was about 11% over water control while in case of joint application of 100 mM NaCl with 50 *μ*M Spm, the chlorophyll content was increased by about 3% over water control.

### 3.3. Effect of NaCl Treatment on the Oxidative Stress Markers

**Figure 6:**
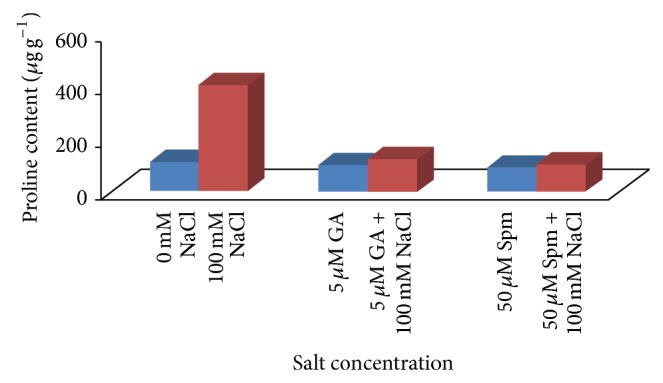
Effect of NaCl on the total proline content of 10-day-old mungbean seedling and its possible amelioration with 5 *µ*M GA_3_ and 50 *µ*M Spm.

**Figure 7:**
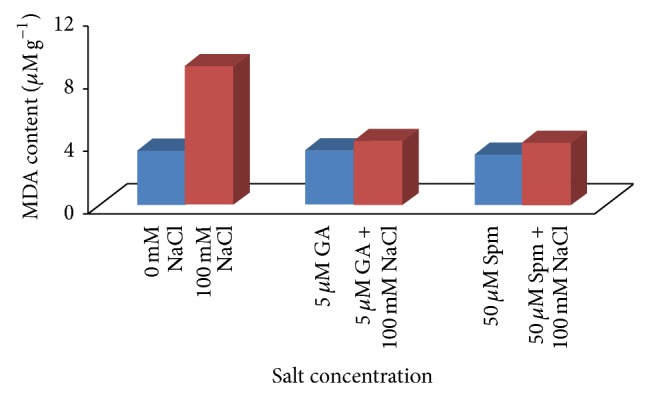
Effect of NaCl on the MDA content of 10-day-old mungbean seedling and its possible amelioration with 5 *µ*M GA_3_ and 50 *µ*M Spm.

**Figure 8:**
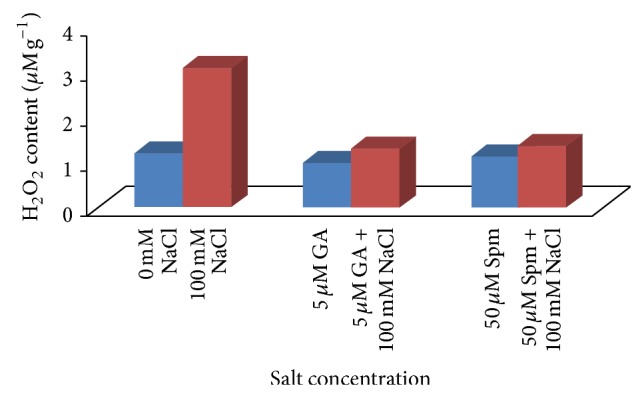
Effect of NaCl on the H_2_O_2_ content of 10-day-old mungbean seedling and its possible amelioration with 5 *µ*M GA_3_ and 50 *µ*M Spm.

Proline, malondialdehyde (MDA), and hydrogen peroxide (H_2_O_2_) contents are utilized as stress markers for oxidative damage and are generally affected by abiotic stress. Proline, an amino acid, acts as a cytoplasmic osmoticum and protects the protein against denaturation [36]. Under salinity stress, osmolyte such as proline maintains cellular homeostasis through osmotic regulation and induces physiological processes favorably. In our experiment, application of 100 mM NaCl concentration resulted in a drastic increase (3.6 times) in the proline content over water control. In case of joint application of 100 mM NaCl with 5 *μ*M GA_3_, the increase in proline content was about 1.1 times over water control, while in case of joint application of 100 mM NaCl with 50 *μ*M Spm, the proline content was almost the same as water control (Figure 6). Malondialdehyde (MDA) which is often used as an indicator of oxidative damage is produced during peroxidation of membrane lipid by decomposition of polyunsaturated fatty acid. In our present study, we observed that MDA content was increased by about 2.5 times at 100 mM NaCl concentrations. In case of joint application of 100 mM NaCl with 5 *μ*M GA_3_, the increase in MDA content was about 1.2 times over water control, while in case of joint application of 100 mM NaCl with 50 *μ*M Spm, the MDA content was increased by about 1.2 times over water control (Figure 7). It has been shown that during salt stress H_2_O_2_ serves as a signal molecule and plays a role in plant defense. Increased level of H_2_O_2_ causes membrane damage due to formation of ROS species. The H_2_O_2_ content was also increased by about 2.6 times at 100 mM NaCl concentration. In case of joint application of 100 mM NaCl with 5 *μ*M GA_3_, the H_2_O_2_ content was almost the same as water control, while in case of joint application of 100 mM NaCl with 50 *μ*M Spm, the H_2_O_2_ content was increased by about 1.1 times over water control (Figure 8). During joint application of either GA_3_ or Spm in low concentration along with NaCl treatment, the inhibitory effect caused by salinity stress was significantly ameliorated over water control in accordance with previously cited literature [2].

### 3.4. Effect of NaCl Treatment on the Antioxidant Enzymes

**Figure 9:**
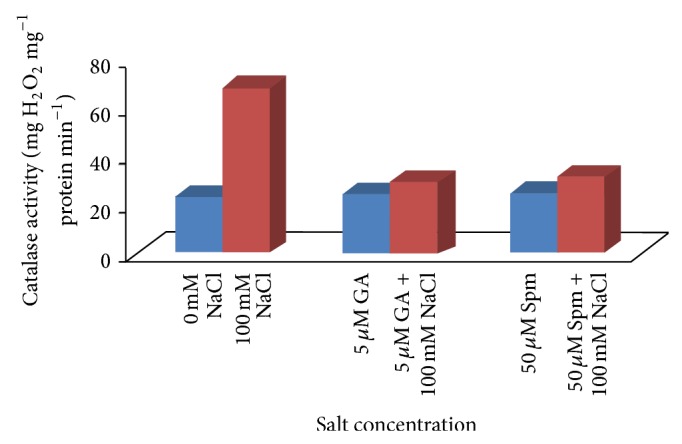
Effect of NaCl on the catalase activity of 10-day-old mungbean seedling and its possible amelioration with 5 *µ*M GA_3_ and 50 *µ*M Spm.

Salt toxicity influences complex biochemical responses and several defensive mechanisms including production of enzymatic as well as nonenzymatic antioxidants, which detoxify ROS that rapidly occurs in plants due to increasing salt concentration. Increased activities of many of the antioxidant enzymes in plants combat oxidative stress induced by salinity stress [9] and various environmental stresses. Maintenance of a high antioxidant capacity to scavenge the toxic ROS has been linked to increased tolerance of plants to these environmental stresses [41, 42]. SOD is a major superoxide scavenger and provides a first line of defense against cellular injury due to abiotic stress. The highly reactive superoxide is then converted to H_2_O_2_ by SOD. The excess H_2_O_2_, which itself is toxic for the plant, is then scavenged by CAT activity. CAT activity was expressed as the amount of H_2_O_2_ decomposed. In our experiment with increase in salinity stress, a significant enhancement in CAT activity (2.9 times) with respect to water control in the seedlings (Figure 9) was observed.

**Figure 10:**
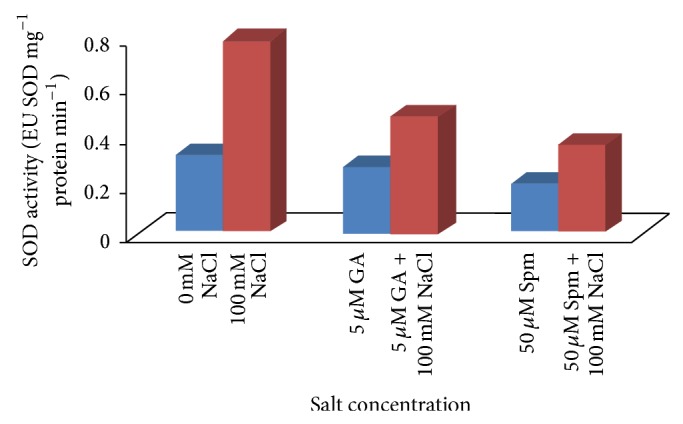
Effect of NaCl on the SOD activity of 10-day-old mungbean seedling and its possible amelioration with 5 *µ*M GA_3_ and 50 *µ*M Spm.

A similar trend was also observed after NaCl stress imposition, where the SOD activity in treated seedlings was drastically elevated (2.4 times) with respect to water control (Figure 10).

Joint application of either GA_3_ or Spm in low concentration along with NaCl in mungbean seedlings altered the activities of antioxidant enzymes in comparison to 100 mM NaCl treatment alone. During joint application of 100 mM NaCl with 5 *μ*M GA_3_, the increment in CAT activity was about 1.2 times over water control, while in case of joint application of 100 mM NaCl with 50 *μ*M Spm, the CAT activity was increased by about 1.3 times over water control (Figure 9). In case of joint application of 100 mM NaCl with 5 *μ*M GA_3_, the increment in SOD activity was about 1.5 times over water control, while in case of joint application of 100 mM NaCl with 50 *μ*M Spm, the SOD activity was increased by about 1.1 times over water control (Figure 10).

## 4. Conclusion

From this present study, it can be concluded that the application of NaCl adversely affected the growth and defense mechanism as well as metabolism of mungbean seedlings. Salt tolerance is a complex phenomenon in plants, and various research methodologies and genetic approaches are used to characterize the diverse biochemical events that occur in response to salt stress. High concentrations of salt induce stunted growth and loss in chlorophyll content as well as oxidative damages by altering antioxidant machinery, leading to membrane damage through lipid peroxidation. Applications of low concentrations of phytohormones such as GA_3_ and PAs such as Spm in presence of NaCl in high concentrations play an antagonistic role in salt uptake. Thus, their use in low concentration in salt contaminated soil may help to grow mungbean plants with normal vigour, better yield and produce salt tolerant varieties.
